# Temperature affects reptarenavirus growth in a permissive host-derived in vitro model

**DOI:** 10.1099/jgv.0.002100

**Published:** 2025-04-29

**Authors:** Iman Kriještorac Berbić, Simon De Neck, Lorenzo Ressel, Eleni Michalopoulou, Anja Kipar, Jussi Hepojoki, Udo Hetzel, Francesca Baggio

**Affiliations:** 1 The BIBD Group and Institute of Veterinary Pathology, Vetsuisse Faculty, University of Zurich, 8057 Zurich, Switzerland; 2 Center for Clinical Studies, Vetsuisse Faculty, University of Zurich, 8057 Zurich, Switzerland; 3 Department of Veterinary Anatomy Physiology and Pathology, Institute of Infection, Veterinary and Ecological Sciences, University of Liverpool, CH64 7TE Neston, UK; 4 Department of Virology, Medicum, Faculty of Medicine, University of Helsinki, 00290 Helsinki, Finland

**Keywords:** Boid inclusion body disease, boid *in vitro *model, growth kinetics, host–pathogen interactions, poikilothermia, reptarenavirus

## Abstract

Reptarenaviruses cause Boid inclusion body disease (BIBD), a lethal disease primarily affecting captive boa constrictors. The presence of cytoplasmic inclusion bodies (IBs), mainly composed of viral nucleoprotein (NP), in various cell types is characteristic to and a diagnostic criterion of BIBD. We have previously reported that reptarenavirus replication and IB formation are efficient in cell cultures that are maintained at 27–30 °C but not in cells that are kept at 37 °C, the temperature commonly used for mammalian cell cultures. Here, considering the poikilothermic nature of snakes, we studied the ideal temperature(s) for reptarenavirus propagation and the expression of reptarenavirus NP. We incubated *Boa constrictor* kidney-derived I/1Ki cells at different temperatures (24–36 °C), inoculated them with University of Giessen virus 1 (UGV-1) and monitored both cell growth and virus proliferation. Cell growth was optimal at 30–34 °C and was not significantly affected by UGV-1 infection. Viral RNA release per cell was highest at ambient temperatures between 28 and 32 °C, as determined by qRT-PCR. However, the cells passaged at day 15 post-inoculation released viral RNA at comparable levels even when kept at slightly lower temperatures (24–26 °C). Morphometric analyses undertaken on sections of cell pellets immunostained for reptarenavirus NP found the expression to be most intense at 32 and 34 °C in freshly inoculated cells, and at 28–32 °C in passaged cells. The NP expression positively correlated with the amount of viral RNA released per cell. Our results indicate that the optimal temperature ranges for boid cell growth and reptarenavirus replication (as judged based on antigen expression and RNA release) overlap at about 32 °C. They also suggest that environmental temperature modulation could represent a strategy to impair reptarenavirus replication and, potentially, the spread of reptarenaviruses within and between snake collections.

## Data Summary

Supplementary material is avaialble with the online version of this article, available through Figshare at https://doi.org/10.6084/m9.figshare.28677704 [1].

**Impact Statement**

Snakes are poikilothermic animals, their body temperature fluctuates with the environmental ambient temperature. Snake viruses, like reptarenaviruses, might therefore have evolved to cope with the continuous changes in the body temperature in their hosts. We have previously shown that reptarenavirus replication is hindered at mammalian body temperatures. Here, using a well-established *in vitro* system, a boa constrictor-derived permanent cell line highly permissive to reptarenavirus infection, we found that the preferential temperature for reptarenavirus growth is around 32 °C, which matches the optimal growing temperature of this cell line and reflects the ambient temperature to which boas are exposed over an extended period of time in their natural habitats. Knowledge of the ideal temperature range for reptarenavirus growth can direct attempts towards limiting reptarenavirus growth in infected animals and spread within captive snake collections.

## Introduction

Boid inclusion body disease (BIBD) is a progressive and usually fatal disease naturally occurring in snakes of the *Boidae* and *Pythonidae* families. BIBD is a worldwide threat to private and zoological collections [2,6], as it mainly affects captive snakes. However, we have recently shown that the disease also occurs in free-ranging boa constrictors in their native regions [7,8]. BIBD is characterized by cytoplasmic inclusion bodies (IBs) in a broad range of cell types in all major organs [2,9,12]. While IBs are the morphological and diagnostic hallmark of the disease, their presence is not associated with overt cytopathic effects [13,14]. In the early 2000s, the IBs were described to consist predominantly of a 68 kDa protein [9]. About a decade later, three independent studies identified arenaviruses (now classified as reptarenaviruses) as the causative agents of BIBD [3,10, 15, 16] and the described 68 kDa protein was identified as the reptarenavirus nucleoprotein (NP) [3,10, 11].

BIBD is associated with a highly variable clinical course. Most striking are central nervous system signs, such as disorientation and incoordination, head tremor, opisthotonos, anisocoria and paresis; more subtle signs are impaired food uptake and regurgitation [2,10, 17, 18]. Clinical disease can vary markedly in severity, at least in boa constrictors; the animals either die within a few months or, more commonly, live for several years with no or only very limited clinical signs [5,6, 13, 17, 19,22]. However, snakes with BIBD often die eventually due to secondary infections manifesting as, e.g. stomatitis, pneumonia or enteritis [11,18], supposedly as a result of immunosuppression [5,9, 10, 17, 23]. This hypothesis is supported by the fact that in BIBD-positive snakes, IBs are abundant in blood precursor cells in the bone marrow and in blood cells [10,12], while anti-reparenavirus antibody levels are low [5,23].

The genus *Reptarenavirus* belongs to the *Arenaviridae* family and *Bunyavirales* order, together with four other genera, *Mammarenavirus*, *Hartmanivirus*, *Antennavirus* and *Innmovirus* [16]. Arenaviruses are enveloped, spherical or pleomorphic viruses with a diameter of 40–200 nm, with a bisegmented (mammarenaviruses, reptarenaviruses and hartmaniviruses) or trisegmented (antennaviruses and innmoviruses) single-stranded ambisense RNA genome [16]. The small (S) and large (L) genome segments of reptarenaviruses encode for two proteins each, the nucleoprotein (NP) and glycoprotein precursor (GPC), as well as the RNA-dependent RNA polymerase (RdRp) and the zinc finger protein (Z), respectively [16]. Often, a genetically diverse swarm of reptarenavirus S and L segments co-exists in individual infected snakes, with L segment species outnumbering the S segments; also, there is evidence that the segment distribution may vary between tissues of an infected individual [4,7,24, 25].

A wide range of viruses, from plant to animal and human viruses, are temperature sensitive [26], likely in association with an adaptation to infection sites. For example, human-adapted respiratory RNA viruses (e.g. coronaviruses, rhinoviruses and influenza virus) grow best at 32–33 °C, the temperatures in the human upper respiratory tract; at higher temperatures, like the 37 °C of the lower respiratory tract or ≥40 °C with fever, their replication rate goes down [26,29]. Reptarenaviruses infect snakes, which are poikilothermic animals; they might therefore have adapted to replicate and grow at fluctuating temperatures. Indeed, we have previously shown *in vitro* that reptarenaviruses replicate effectively not only in boa constrictor (I/1Ki), i.e. reptilian, but also in mammalian and arthropodian cell lines when maintained at 27–30 °C, as indicated by viral NP accumulation and IB formation [10,30]. Albeit, viral RNA levels and NP expression markedly decreased when we maintained the cells at 37 °C, suggesting reptarenavirus growth inhibition at mammalian body temperature [30]. However, in that study, focusing on assessing the risk of cross-species transmission, we did not elucidate the temperature range for optimal growth of reptarenaviruses. We have now addressed the latter with an in-depth investigation, using a single reptarenavirus isolate (University of Giessen virus 1; UGV-1) to infect our well-established I/1Ki boid cell line incubated at temperatures ranging from 24 to 36 °C. We used quantitative real-time PCR (qRT-PCR), immunocytochemistry (ICC) and morphometric approaches to quantify viral growth and NP expression in cells that were either freshly infected or passaged once after earlier inoculation, to determine the optimal temperature range for reptarenavirus life cycle completion.

## Methods

### Cell culture and virus isolate

The permanent I/1Ki cell line, derived from the kidney of a juvenile *Boa constrictor* [10], was maintained in Minimum Essential media (MEM, Gibco, Thermo Fisher Scientific, Waltham, MA, USA) supplemented with 10% fetal bovine serum (FBS, Biochrom, Cambridge, UK), 10% tryptose phosphate broth (TPB, Difco, Franklin Lakes, NJ, USA), 6 mM Hepes (Biochrom), 2 mM l-alanyl-l-glutamine (Seraglob by Bioswisstec AG, Schaffhausen, Switzerland) and 50 µg ml^−1^ Gentamicin (Gibco, Thermo Fisher Scientific). The standard conditions for maintenance are incubation at 30 °C and 5% CO_2_ [10].

For virus inoculations, University of Giessen virus 1 (UGV-1, respective S and L segment GenBank accession nos. KR870012 and KR870022) [25] was used. A stock with 5×10^8^ 50% Tissue Culture Infective Dose (TCID50) ml^−1^ (immunofluorescence (IF)-based end-point dilution assay, see below) was prepared as previously described [31].

I/1Ki cells were seeded onto two 6-well plates (7.5×10^5^ cells per well) and six T75 cm^2^ flasks (6.2×10^6^ cells per flask) (TPP, Trasadingen, Switzerland) per temperature (see below) 1 day before virus inoculation (D−1), to yield an equivalent cell density of approximately 83,000 cells cm^−2^ and approximately 30% confluency. They were placed in incubators adjusted to the following temperatures: 24, 26, 28, 30, 32, 34 or 36 °C. The following day (D0), the cells were inoculated with UGV-1 at a multiplicity of infection (MOI) of 10 or mock infected (media only), allowing the virus to adsorb for 2–3 h at the respective temperature. Cells were washed twice with media and incubated at different temperatures for 3 days at which point (D3) one set of plates and flasks was processed for further examinations. Supernatants were collected and media replaced in a second set of wells and flasks; the former were processed for further examinations at D6. The third set of infected flasks was incubated for 15 days, with media changes every 3 days, and then passaged (P1), following trypsinization, onto new 6-well plates (7.5×10^5^ cells per well) or T75 cm^2^ flasks (6.2×10^6^ cells per flask).

The experiments on 6-well plates were run in triplicates, whereas a single infected and mock-infected T75 cm^2^ flasks were prepared for each temperature and timepoint.

The cells were counted using the LUNA-II Automated Cell Counter (Logos Biosystems, Dongan-gu Anyang-si, Gyeonggi-do, South Korea). The supernatants from the 6-well plates were collected and stored at −80 °C for RNA isolation. The cells from the T75 cm^2^ flasks were pelleted and processed for ICC.

The passaged cells (P1) were re-counted 1 day after seeding to assess the number of adherent cells, comparable to what was done with the naïve cells (P0). This was followed by cell counting, collection of the supernatant and pellet preparation 3 and 6 days later, following the same protocol as for the freshly infected cells.

### End-point dilution assay for virus titration

To quantify the infectious particles in the UGV-1 stock, a 10-fold dilution series (from 10^−1^ to 10^−12^) of the virus stock was prepared, and the diluted virus was dispensed onto approximately 80% confluent I/1Ki cells on a 96-well plate (8 wells per dilution). At 5 days post-inoculation (dpi), the cells were fixed with 4% paraformaldehyde (PFA) in phosphate-buffered saline (PBS) for 15 min at room temperature (RT). After a PBS wash, the cells were permeabilized and blocked with 0.25% Triton-X-100 (Sigma-Aldrich, Merck, Darmstadt, Germany) and 0.5% (w/v) bovine serum albumin (BSA, Sigma-Aldrich, Merck) in PBS for 10 min at RT. After two washes with PBS, the cells were incubated overnight at 4 °C with a rabbit anti-pan-reptarenavirus NP serum [7] diluted 1 : 6,000 in Dako REAL antibody diluent (Agilent Technologies, Santa Clara, CA, USA). This was followed by three PBS washes, incubation with Alexa Fluor 594 goat anti-rabbit (Thermo Fisher Scientific) diluted 1 : 400 in Dako REAL antibody diluent for 1 h at RT and then three washes with PBS. Cells were finally incubated for 15 min at RT with 100 ng ml^−1^ DAPI (Novus Biologicals, Littleton, CO, USA) in PBS, followed by two washes with milli-Q water. Afterwards, for each dilution, the wells presenting at least one fluorescent focus forming unit (FFFU) were counted using a Nikon Eclipse TI microscope (Nikon, Tokyo, Japan) and the TCID50 ml^−1^ was determined using the calculator provided by Marco Binder (available via https://www.klinikum.uni-heidelberg.de/fileadmin/inst_hygiene/molekulare_virologie/Downloads/TCID50_calculator_v2_17-01-20_MB.xlsx), as previously described [32].

### RNA isolation and qRT-PCR

The QIAamp Viral RNA Mini Kit (Qiagen, Venlo, The Netherlands) used with carrier RNA according to the manufacturer’s instructions served for RNA extraction of the cell culture supernatants stored at −80 °C. The RNAs were eluted twice with 40 µl of nuclease-free water (AppliChem, Darmstadt, Germany), then stored at −80 °C.

We used qRT-PCR primers (Microsynth AG, Balgach, Switzerland) and probe (Metabion International AG, Planegg, Germany) targeting the UGV-1 S segments for quantifying the UGV-1 S segment RNA released into the cell culture supernatants by the UGV-1 infected cells, applying a recently described qRT-PCR protocol [33]. Tests were run in duplicates on MicroAmp Fast Optical 96-well reaction plates (Applied Biosystems, Thermo Fisher Scientific) with the 7500 Fast real-time PCR system and software v2.0 (Applied Biosystems).

### ICC for reptarenavirus NP

UGV-1-infected and mock-infected I/1Ki cells in T75 cm^2^ flasks were trypsinized (0.25% Trypsin-EDTA 1×, Gibco, Thermo Fisher Scientific, Waltham, MA, USA) for 5 min at RT, collected and pelleted through centrifugation for 5 min at 1,000×*g* at 10 °C. The cell pellets were fixed in 4% PFA (pH 7.4) for 24 h and routinely paraffin wax embedded. Ten (or five when the pellets were too small to allow more) consecutive sections (3–5 µm) were prepared and subjected to immunocytochemical staining for reptarenavirus NP, using a previously described protocol [10] and a previously reported rabbit anti-pan-reptarenavirus NP serum (dilution 1 : 6,000) [7].

### Digital image analyses

The slides with sections of the cell pellets stained for reptarenavirus NP by ICC were scanned using the NanoZoomer 2.0 HT (Hamamatsu, Hamamatsu City, Japan) at ×40 magnification. Whole slide images (WSI) were imported into Aperio ImageScope (v12.4.6.5003; Leica Biosystems, Nussloch, Germany) to separate the pellets into individual section files. Afterwards, three digital image analysis approaches were used on the output files to determine the percentage of NP immunostained area and the percentage of NP positive cells, respectively.

For two of the three approaches, the individual sections were imported to a QuPath project (v0.5.1, https://qupath.github.io/; [34]) and were manually annotated to define regions of interest (ROIs), excluding areas with artefacts or with non-specific staining outside the pellet section area (Fig. S1. available in the online version of this article). The manual annotation was refined using a pixel classifier in order to exclude the background and keep only the cell pellet area. Four sections were used to train a pixel classifier to account for variations in 3,3′-diaminobenzidine (DAB) staining. Manual annotations defined ‘Pellet’ (positive/negative cells) and ‘Ignore’ (background) classes. The classifier used a built-in artificial neural network (ANN_MLP) at 0.91 µm/px resolution. The refined ROIs were passed to a second-pixel classifier, using a threshold on the DAB channel (threshold set at 0.24) at the same resolution (0.91 µm/px), to classify each pixel as either positive or negative.

A similar approach served for quantifying the percentage of infected cells, with the exception that Cellpose (v3.09, https://github.com/MouseLand/cellpose; [35]) via the Cellpose extension for QuPath (v0.9.0; [34,36,39]) served for cell segmentations, using Anaconda (Anaconda3 v2024.02-1, Anaconda Inc., Austin, TX, USA) with graphics processing unit (GPU) acceleration (pytorch v2.3.1, pytorch-cuda v12.1) on a Nvidia RTX A5000 GPU (Nvidia, Santa Clara, CA, USA) (Fig. S1). Briefly, Cellpose was run on the manually annotated pellet sections, using the built-in ‘cyto3’ model to perform cell segmentation (pixelSize = 0.5; channels = 0; tileSize = 1024). The section with the segmented cells was then subjected to an object classifier to classify the cells as either positive or negative, using a threshold method on the DAB channel (threshold = 0.24; measurement: DAB: Max).

Afterwards, following a visual quality control step, the percentage of NP positive area or positive cells (%) was calculated as the ratio between [NP positive area (µm^2^)]/[total area (µm^2^)]) × 100 or [NP positive cells]/[total number of cells]) × 100, respectively.

A third tool based on a convolutional neural network (CNN) semantic segmentation model for pixel-level characterization of staining/subcellular areas (cytoplasm and nucleus) was developed in parallel (Fig. S1). For training, the ground truth was created by manually annotating regions corresponding to classes of interest (Background, Nuclei, Positive Cytoplasm, Negative Cytoplasm) on 10 randomly chosen sections. ‘Negative cytoplasm’ was considered when no DAB staining was observed. ‘Positive cytoplasm’ was considered when at least a brown dot was visualized in a cell. Each class had at least 200 examples annotated.

Annotated WSI were imported into MIMPro (Medical Image Manager Pro; HeteroGenius). The deep-learning process took approximately 2 days on a system equipped with 2× Nvidia Quadro RTX6000 GPUs (Nvidia) MIMPro with Deep Learning Addon (Medical Image Manager Pro with Deep Learning Add On; HeteroGenius). The employed CNN architecture was UNet [40]. The network was trained for a total of 2,000 epochs on the training set with batch sizes of 32. Patches of 512×1024 pixels and ×400 magnification were used. Random transforms (image augmentation) were used to increase generalisation and improve the algorithm’s robustness for variation in cell morphology and staining intensity. Validation of the CNN was performed on another set of stained sections not used for training. Average Accuracy, Mean Precision, Mean Recall, Mean Specificity and Mean F1 were 0.93, 0.94, 0.93, 0.98 and 0.93, respectively. The model obtained was deployed through MIMPro on all sections (*n* = 250) to create a segmentation mask with individual pixels assigned to one of the pre-defined classes (semantic segmentation). The masks were then imported into a QuPath project (v0.5.1, https://qupath.github.io/; [34]), converted to the OME-TIFF format (without downsampling) with the pixel size (µm) embedded, imported into Visiopharm 2024.07.1.16850×64 RC (Visiopharm, Hoersholm, Denmark) and the ROI defined manually as described above. Quantification of each of the classes created by the CNN was achieved by running an Analysis Protocol Package (APP) based on a decision Forest method; briefly, manual annotations of the four classes were drawn and served to train the APP to recognise them. Once the analysis was completed, a visual quality control was performed on all sections to confirm that the APP ran properly. The percentage of NP positive area (%) was calculated as the ratio between the NP positive cytoplasmic area and the total area, according to the following formula: ([NP positive cytoplasmic area (µm^2^)]/[nuclei area +NP negative cytoplasmic area +NP positive cytoplasmic area (µm^2^)]) × 100.

### Statistical analyses

Statistical analyses were performed to examine the associations between measured (cell growth, UGV-1 S segment RNA levels and NP expression) and defined parameters (temperature, time after virus inoculation and passage number). Statistical analyses on the cell number counts were performed using Stata13 (Stata statistical software: release 13, 2013; StataCorp LLC, College Station, TX, USA) and pairwise mean comparisons were performed using the Bonferroni correction. The statistical analyses on UGV-1 S segments released per cell and on NP expression were performed in R (v4.4.0 (2024-04-24 ucrt) [41]), using RStudio (RStudio: Integrated Development Environment for R, version 2024.09.1 Build 394, Posit Software, PBC, Boston, MA, USA) with the following packages: ggpubr (0.6.0), smplot2 (0.2.4), tidyr (1.3.1), stringr (1.5.1), rstatix (0.7.2), magicfor (0.1.0), ggplot2 (3.5.1), FSA (0.9.5), dplyr (1.1.4), car (3.1-2), carData (3.0-5) and agricolae (1.3-7). The data collected from each individual condition, i.e. a specific timepoint after virus inoculation, at different temperatures and passage (P0, P1), originated from three biological replicates for the qRT-PCRs and 10 (or 5) technical replicates (i.e. sections stained for NP obtained from a single cell pellet) for the quantification of NP expression. A Shapiro–Wilk test was performed to test for normality of the data. When data followed a normal distribution, i.e. for the qRT-PCR results, the equality of the variances was evaluated either through a Fisher’s F-test for two group comparisons or through a Bartlett test, when comparing multiple (>2) groups. The outcome of non-equal variances for some of the comparisons of each test resulted in a Welch two-sample *t*-test for the comparison pairs, and in a one-way Welch’s ANOVA using a Games–Howell post hoc test for the multiple comparisons. Alternatively, in the case of non-normal data distribution, i.e. NP expression in pellet sections, a Mann–Whitney *U* test (Wilcoxon rank sum exact test) was performed for all pair comparisons, whereas a Kruskal–Wallis test using Dunn’s post hoc test with *P*-values adjusted with the Bonferroni method was performed for the multiple comparisons. In addition, using the averaged values of the replicates of each condition, the correlation between the NP measurements obtained through the three quantification methods as well as the correlation between the viral RNA released per cell with the NP positive areas/cells in pellet sections were assessed with Spearman’s rank correlation, as not all comparison groups could be considered to follow a normal distribution (Shapiro–Wilk test). The level of significance of all the applied statistical tests was at 5% (95% confidence interval).

## Results

### I/1Ki cell growth is optimal at 30–34 °C and not significantly affected by UGV-1 infection

**Fig. 1.**
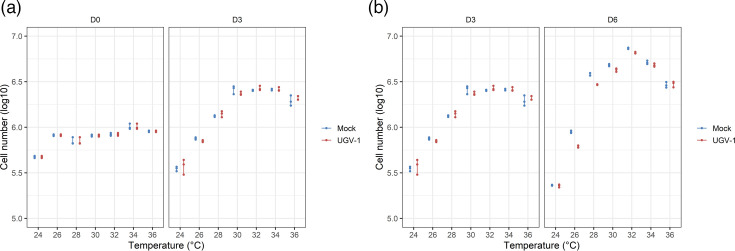
Growth of mock-infected and freshly UGV-1 infected I/1Ki cells. I/1Ki cells were seeded onto 6-well plates at 7×10^5^ cells/well and incubated at different temperatures (24–36 °C). The adherent cells/well were counted with a LUNA-II Automated Cell Counter at D0 (1 day after seeding) and at D3 and D6, after inoculation with UGV-1 (MOI = 10) or mock inoculation at D0. We used biological triplicates for each condition. The figure shows scatter plots of the cell numbers (*Y*-axis, logarithmic scale) at the different temperatures and timepoints (*X*-axis), comparing D0 and D3 (**a**) and D3 and D6 (**b**). These data are separated into two graphs to ease the comparison of Fig. 1b to the graphic representations of the subsequent data in Figs 2 and 4, composed only by D3 and D6 collections. The raw data are provided in Table S1. The statistical analyses are presented in Figs S2, S3 and S5, and Table S2.

The I/1Ki boa constrictor kidney derived cells are well established as a tool for reptarenavirus isolation and growth [8,10, 14, 30]. As a pre-requisite to determine the optimal temperature range for reptarenavirus replication, we examined the ambient temperature range that best supports I/1Ki cell growth. Mock-infected I/1Ki cells were seeded onto 6-well plates and incubated at temperatures between 24 and 36 °C, at 2 °C intervals. We quantified the adherent (i.e. viable) cells 1 day after seeding (D0), before proceeding with UGV-1 inoculation for a subset of cells. In addition, we counted both the adherent mock-infected and the freshly infected cells another 3 days (D3) and 6 days later (D6, after a media change at D3). On D0, the number of cells that had adhered was similar for all temperatures, except for 24 °C, where the number was on average almost 50% lower (Fig. 1a).

At D3 and D6, cell numbers obtained from mock-infected samples incubated at temperatures between 28 °C and 34 °C suggested that the cells were continuously dividing during the entire study period (Fig. 1b). With an ambient temperature of 24 °C, we observed a statistically significant decrease in the cell quantity over time, whereas at 26 °C cell numbers appeared to remain static. This suggests impaired cell growth at temperatures ≤26 °C. At 36 °C, we observed a significant increase in the cell numbers from D0 and D3; however, the number had not significantly increased by D6 (Fig. 1b). A closer look at the 28–34 °C range indicated the fastest I/1Ki cell growth between 30 and 34 °C; at 28 °C, the cell numbers were lower at both D3 and D6 when compared to 30, 32 and 34 °C, although the difference was statistically significant for the three temperatures at D3 only. Quantification of cell numbers after infection with UGV-1 revealed no significant differences in comparison to the mock-infected cells. The results are illustrated in Figs 1 and S2 and S3, and shown in detail in Tables S1 and S2.

Assessment of the cells reseeded 15 days after UGV-1 inoculation (P1) for growth yielded cell counts comparable to those obtained at D3 and D6 in P0 (i.e. mock-infected and freshly UGV-1 infected cells), with a peak in cell growth at 30–34 °C. Interestingly, at 24 °C, P1 cell numbers remained static over time but did not decrease like in mock-infected and freshly infected P0 cells. The results are illustrated in Figs S2–S5 and shown in detail in Tables S1 and S2.

Since we planned to use cells grown on T75 cm^2^ flasks to generate cell pellets for the ICC approach to evaluate viral protein expression, we repeated the above experiments with cells grown in T75 cm^2^ flasks, with one flask per temperature and timepoint. This yielded results comparable to those obtained from cells seeded onto 6-well plates (Fig. S6; Table S1).

Taken together, the results suggest that UGV-1 infection does not affect I/1Ki cell growth over the studied temperature range of 24–36 °C.

### Viral RNA release is highest at 28–32°C

We have previously established that quantification of viral genomic RNA released by infected cells reflects reptarenavirus replication [33]. Hence, our earlier observation of a strong reduction in the release of viral genomic RNA from infected I/1Ki cells incubated at 37 °C in comparison to 30 °C [30] can be considered as evidence of the temperature sensitivity of reptarenaviruses. Here, we aimed to identify the optimal temperature range for reptarenavirus proliferation and wanted to determine whether it overlaps with the one for I/1Ki cell growth.

Therefore, we collected samples of the cell culture media at 3 day intervals, from both the freshly infected cells (P0) and the cells passaged at 15 dpi (P1). We employed qRT-PCR to quantify the number of UGV-1 S segments secreted per ml of media. This allowed us to determine the number of segments secreted per well over each 3 day interval, as well as the cumulative number of segments secreted per well for D6 (i.e. the sum of the values obtained at D3 and D6, given that media was replaced at D3). We then calculated the (cumulative) number of segments secreted per cell by normalizing the values to the corresponding cell counts, to evaluate the effect of temperature on viral release independently from the influence of temperature on cell growth (Table S3).

**Fig. 2.**
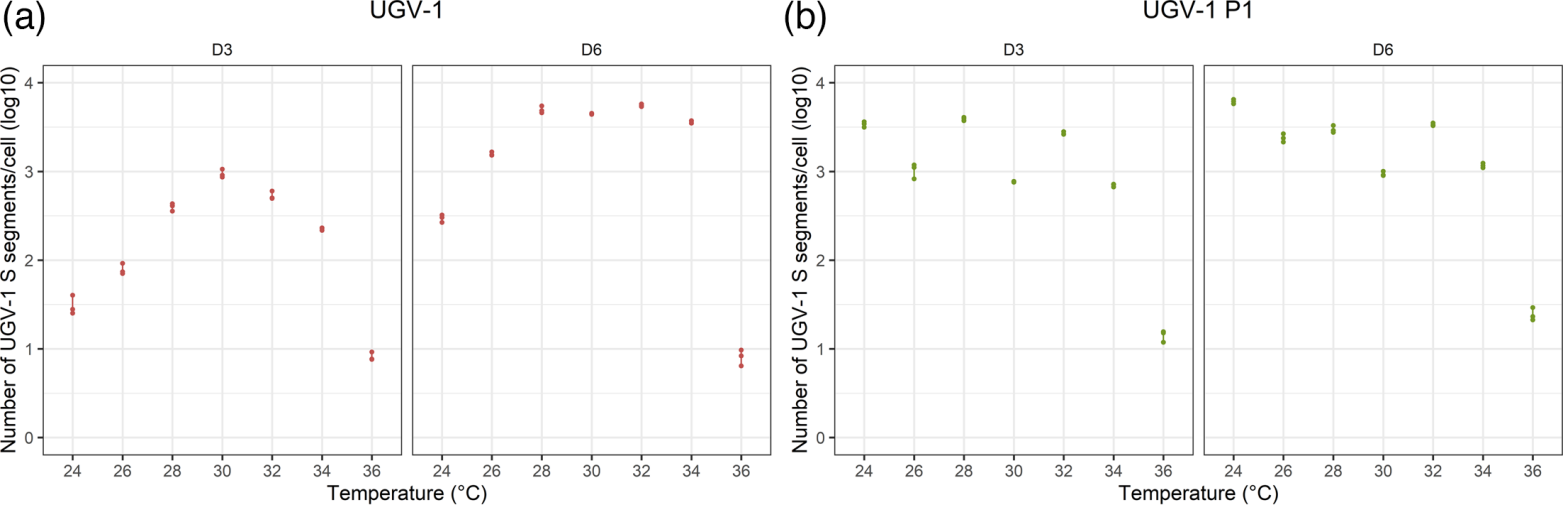
Viral RNA release per infected I/1Ki cell. Viral RNA was isolated from I/1Ki cell culture supernatants collected at D3 and D6 after UGV-1 inoculation (MOI = 10) and incubation at 24–36 °C, and at the corresponding timepoints after passaging I/1Ki cells at 15 days post-UGV-1 inoculation (P1). The number of UGV-1 S segments released per cell was quantified through qRT-PCR from biological triplicates for each condition. The figure shows scatter plots of the number (cumulative for D6) of UGV-1 S segments released per cell (*Y*-axis, logarithmic scale) for freshly UGV-1 inoculated cells (**P0, a**) or cells passaged at 15 days post-UGV-1 inoculation (**P1, b**) at the different timepoints and temperatures (*X*-axis). The raw data are provided in Table S3. The statistical analyses are presented in Figs S7, S8 and S10, and Table S4.

For freshly infected cells, the (cumulative) number of UGV-1 S segments/cell secreted into the media ranged from 8 to 943 at D3 and from 8 to 5,559 at D6. For D3, the number was highest for cells kept at 30 °C, with almost 1,000 s segments/cell, with a significant difference compared to all other temperatures. The second highest numbers were obtained at 28 and 32 °C; these were significantly higher than those of the remaining temperatures. For D6, the cumulative number was significantly highest between 28 and 32 °C, with >4,400 s segments/cell. Cells kept at either 24 °C or 36 °C showed the significantly lowest numbers at both D3 and D6. The results are illustrated in Figs 2 and S7 and shown in detail in Tables S3 and S4. For all temperatures between 24 and 34 °C, the cumulative numbers at D6 lay above the values at D3. Indeed, from D3 to D6, the number of viral segments secreted per cell increased between 4 and 18.7 times compared to the increase from D0 to D3. In contrast, at 36 °C, the number of segments secreted per cell from D3 to D6 was almost null, leading to comparable cumulative D3 and D6 values (Figs S8 and S9; Table S3).

For the cells passaged at 15 dpi and kept at the indicated temperatures, the (cumulative) amount of UGV-1 S segments/cell in the media collected at D3 varied from 14 to 3,910 copies/cells; at D6, it was slightly higher (24–6,140 copies/cell). At D3, the highest numbers were obtained from cells kept at 24, 28 and 32 °C, with >2,600 copies per cell; the difference was statistically significant compared to all other temperatures and, at D6, this also applied to cells kept at 26 °C (≥2,400 copies/cell). Different from the freshly infected cells, also cells kept at 24 and 26 °C demonstrated secretion of UGV-1 S segment RNA. However, it cannot be excluded that the passaged cells adhered better than the freshly infected cells because they might have adapted to the lower temperature; this could have influenced the result. Interestingly though, the P1 cells showed the lowest amount of viral S segments in the cell culture supernatants when kept at 36 °C. The results obtained from the P1 cells are illustrated in Figs 2 and S7, S8, and in detail in Tables S3 and S4. They showed the amount of UGV-1 S segment RNA released/cell per 3-day interval to peak at D3 for cells kept at 24 °C and 28–32 °C, and to slightly decrease afterwards, from D3 to D6 (non-cumulative number), while it slightly increased in the case of cells kept at 26 °C and remained stable for cells kept at 34–36 °C (Fig. S9; Table S3). Differently from freshly infected cells, P1 cells incubated at all different temperatures released an approximately constant amount of viral genome over time.

The comparison of (cumulative) viral RNA copy release per freshly infected cell and P1 cell showed a significantly higher number for the latter at the D3 sampling timepoint; at D6, this only applied to cells kept at 24, 26 and 36 °C, whereas incubation between 28 and 34 °C resulted in significantly higher numbers for the freshly inoculated cells (Figs 2 and S10; Tables S3, S4). Performing the experiment on cells grown in T75 cm^2^ flasks yielded similar results (data not shown). To conclude, the results suggest that the ideal reptarenavirus growth temperature is between 28 and 32 °C.

### Viral NP expression is most intense at 32 and 34°C in freshly infected cells and at 28 and 32°C in passaged cells

In parallel to the quantification of genomic RNA in the cell culture supernatants, we examined the viral protein expression in I/1Ki cells freshly UGV-1 inoculated or passaged at 15 dpi, taking a well-established immunocytochemical approach that detects reptarenavirus NP (and thereby also the typical IB) in formalin-fixed, paraffin-embedded pellets prepared from the cultured cells [10,30]. To obtain sufficient cells for cell pellet preparation, we used the cells grown in T75 cm^2^ flasks.

Expression of NP in the I/1Ki cells was represented by a focal cytoplasmic reaction that generally emphasised the IBs [10,30]; the staining pattern and extent are illustrated in Fig. S11.

**Fig. 3.**
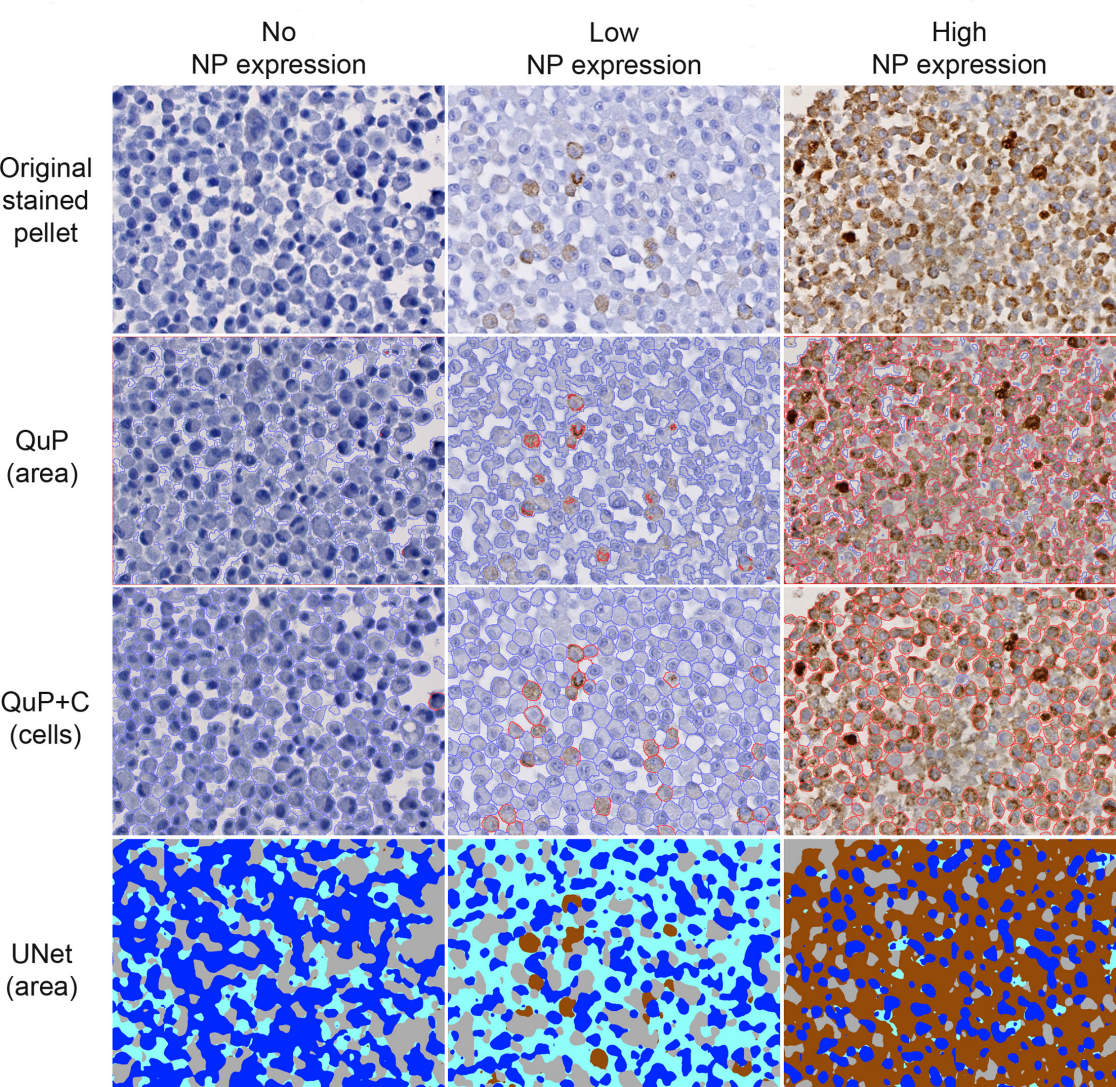
Positive cell selection using QuP, QuP+C and UNet on UGV-1 infected I/1Ki cell pellet sections stained for viral NP. Pellet sections were immunostained using the anti-reptarenavirus NP antiserum and DAB as chromogen, followed by haematoxylin counterstain. Three examples with either absent (left column), low (middle column) or high (right column) NP expression are presented and include the original immunostained sections (top row) or segmentation and classification outputs by QuP (second row; NP positive area: red outline; NP negative area: blue outline), QuP+C (third row; NP positive cells: red outline; NP negative cells: blue outline) or UNet (bottom row; NP positive cytoplasm/area: brown; NP negative cytoplasm/area: pale blue; nuclei: dark blue; background: grey).

For unbiased quantification of NP expression, three digital image analysis tools were employed (Figs 3 and S1), (a) the QuPath software [34] (now called ‘QuP’), (b) QuPath with the Cellpose software using a built-in cytoplasmic segmentation AI model [35] (now called ‘QuP+C’) and (c) UNet generated masks (now called ‘UNet’). QuP allowed to segment and classify the area covered by the cells into regions either positive or negative for NP, while QuP +C allowed cell segmentation and classification as either positive or negative for NP. For UNet, small, highly representative subsets of immunostained sections served to train the algorithm by allocating the cell (structures) to four classes: (*i*) nuclei; (*ii*) NP positive cytoplasm; (*iii*) NP negative cytoplasm; (*iv*) background. Although QuP and UNet both measure the positive area for NP, the way they detect the DAB precipitate differs. While QuP uses a manually defined colour intensity threshold to classify each pixel as being from a NP positive region or not, UNet determines if a given pixel is part of the NP positive cytoplasm class, based on complex patterns learnt by examples on labelled areas, not necessarily depending on staining intensity. Therefore, these two methods assess the same parameter (NP positive area) but at a different level of abstraction. The quantification outputs of all three methods were converted to the percentage of NP positive area (QuP and UNet) or NP positive cells (QuP +C).

**Fig. 4.**
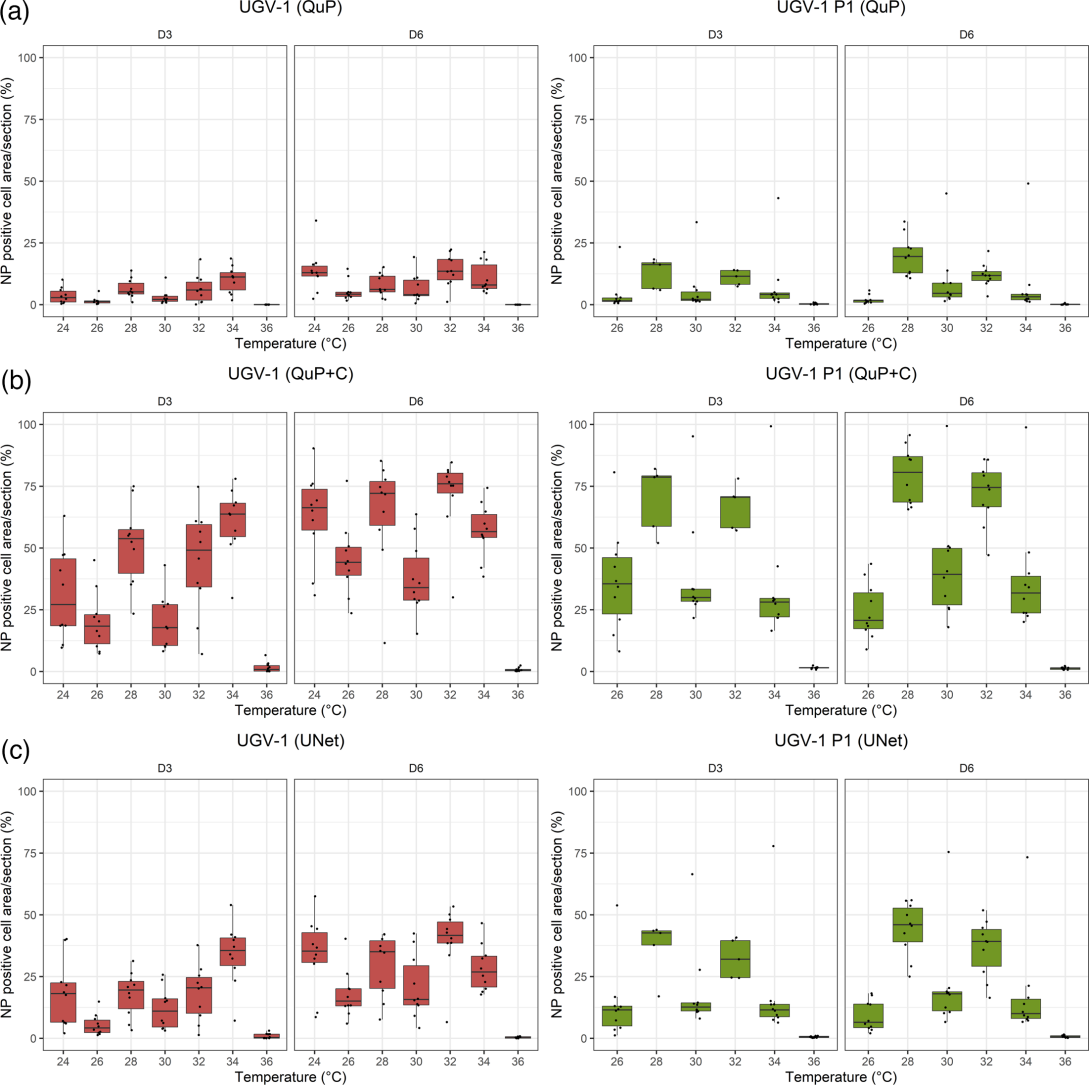
Quantification of NP expression in UGV-1 infected I/1Ki cell pellet sections stained for viral NP. Cell pellets were prepared, formalin-fixed and paraffin-embedded. For each pellet, 10 (5 if pellets too small) consecutive sections were immunostained using a rabbit antiserum raised against reptarenavirus NP and DAB as chromogen, with haematoxylin counterstain. The figure shows box-plots overlaid with scatter plots of the percentages of NP-positive (**a**) cell area/section determined by QuP, (**b**) cells/section determined by QuP+C, (**c**) cell area/section determined by UNet (*Y*-axis, logarithmic scale) for freshly UGV-1 inoculated cells (UGV-1) or cells passaged at 15 days post-UGV-1 inoculation (UGV-1 P1) at the different timepoints and temperatures (*X*-axis). The raw data are provided in Table S5. The statistical analyses are presented in Figs S12–S14 and Table S4.

In freshly infected cells at D3, the extent of NP expression was highest in cells kept at 34 °C; here, an average of 60.6% of cells was found to be positive (QuP+C analysis), and the NP positive area was determined as 10.2% (QuP analysis), and 33.92% (UNet analysis), respectively. By D6, the values had consistently increased for cells maintained between 24 and 32 °C (Figs 4 and S12, S13; Table S5). However, at this point, they were highest for cells kept at 32 °C (QuP+C: 71.7% NP positive cells; 13.9% (QuP) and 39.66% (UNet) positive area). For P1 cells, both at D3 and D6, the three methods identified the highest NP expression values in cells kept at 28 and 32 °C (67–79.3% positive cells (QuP +C); 11–19.7% (QuP) and 32.29–44.19% (UNet) positive area). Also, in P1 cells, the values did not vary significantly between the timepoints (Figs 4 and S13). The lowest values were obtained for both freshly inoculated and P1 cells maintained at 36 °C. However, between 24 and 34 °C, in both freshly infected and P1 cells, the values followed the temperature gradient in a somewhat discontinuous manner. The detailed results are provided in Tables S4 and S5, and the graphical illustration is provided in Figs 4 and S12–S14.

In comparison to the freshly infected cells, for P1 cells, the analyses indicated similar or increased NP positive percentage area/cells between 28–32 °C and a tendency of lower NP positive area/cells at 34 °C at both time-points, and at 26 °C at D6 only (Figs 4 and S14).

The three analytic approaches showed very strong and statistically significant (*P*-values < 0.001) positive correlations, with a Spearman’s rank correlation coefficient (*R*) for each comparison ≥0.9 when considering P0 and P1 individually or together (Figs S15 and S16).

To conclude, the results showed the highest NP expression in freshly inoculated cells at D3 when kept at 34 °C, and at D6 when kept at 32 °C. In P1 cells, the highest amount of NP was detected in cells maintained at 28 and 32 °C at both D3 and D6. Interestingly, the extent of NP expression in the cells seemed not to follow the temperature in a linear fashion, given that it was higher at 24 °C versus 26 °C, and at 28 and 32 °C versus 30 °C. In cells maintained at 36 °C, NP expression was barely detectable, which aligned with the low release of viral genomic RNA, indicating severe inhibition of viral replication.

### NP expression positively correlates with viral RNA release

**Fig. 5.**
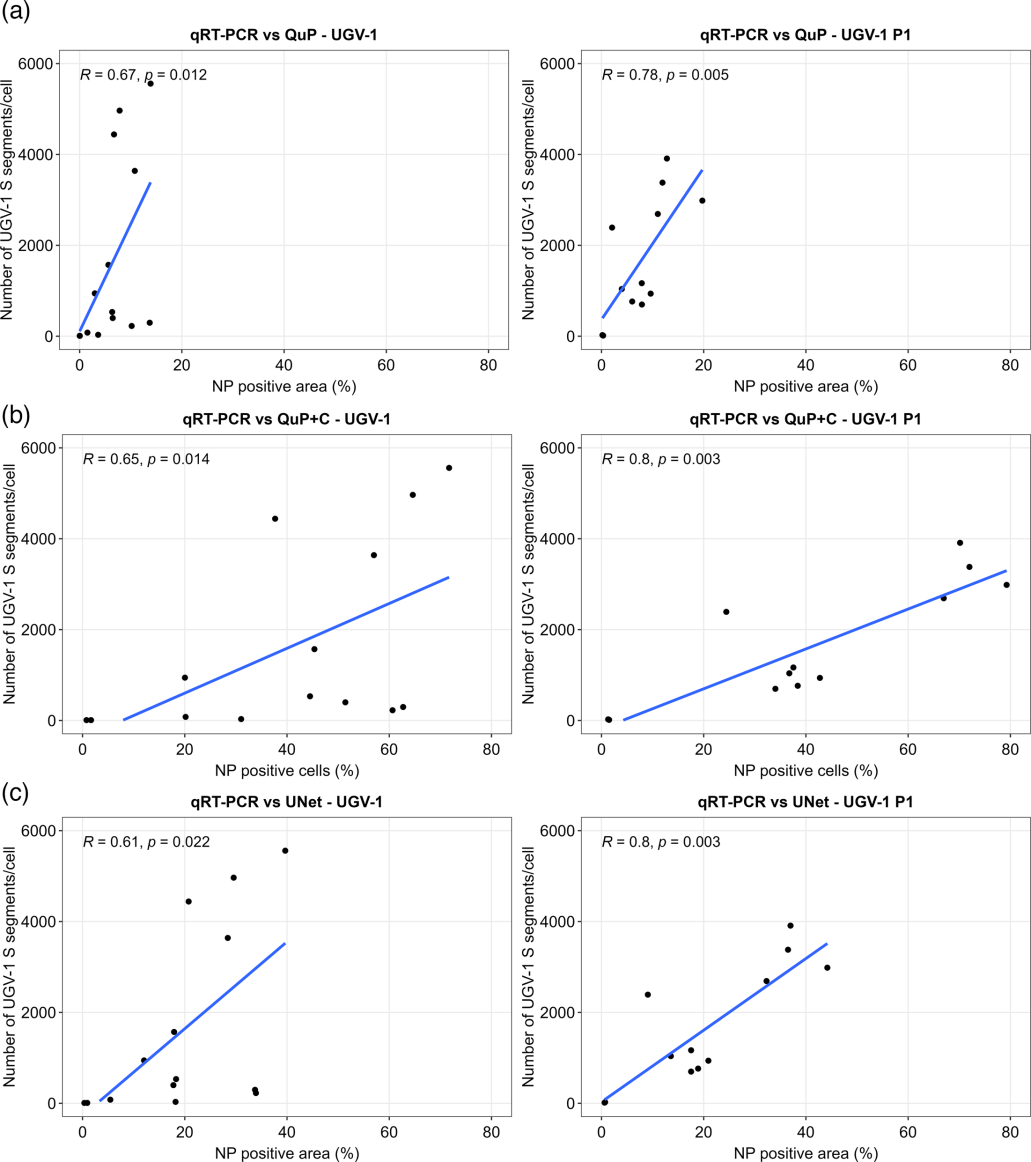
In both passages, NP expression positively correlates with viral RNA release. We calculated the Spearman’s rank correlation coefficient (*R*) for the comparisons between the number of UGV-1 S segments released/cell vs the percentage of NP-positive (**a**) cell area/section determined via QuP; (**b**) cells/section determined via QuP+C; (**c**) cell area/section determined via UNet. The comparisons were made using either freshly UGV-1 inoculated cells (UGV-1) or cells passaged 15 days post-UGV-1 inoculation (UGV-1 P1). The *R* and *P*-values are shown in the respective graphs and in Fig. S16.

Finally, we performed statistical correlation analyses to establish whether reptarenavirus release/cell and the amount of NP expression follow the same trend in relation to temperature, timepoint and passage. The results from both freshly infected (P0) and passaged (P1) cells showed a statistically significant positive correlation between NP expression and the release of viral RNA into the media. Specifically, we observed a stronger positive correlation for P1 than for P0, with *R* between 0.78 (*P* = 0.005) and 0.8 (*P* = 0.003) in P1 and between 0.61 (*P* = 0.022) and 0.67 (*P* = 0.012) in P0, depending on the method used for quantification of NP expression (Figs 5 and S16).

## Discussion

Inspired by our earlier observation that reptarenavirus growth is impaired at 37 °C [30], the present study investigated the effect of temperature in the same *in vitro* system, i.e. on reptarenavirus proliferation and NP expression (as a surrogate for IB formation) as well as on the boa constrictor cells used to grow the virus. We employed I/1Ki cells, a spontaneously immortalized *Boa constrictor* kidney-derived cell line [10], and determined all parameters after the cells had been maintained at the temperature range between 24 and 36 °C for 1, 4 or 7 days. The cells grew at temperatures between 28 and 36 °C, although the optimal temperature appeared to be between 30 and 34 °C, perhaps reflecting the poikilothermicity of boa constrictors. Inoculation with UGV-1, a reptarenavirus isolate [25,42], did neither affect the growth of freshly inoculated I/1Ki cells nor of cells passaged 15 days post-UGV-1 inoculation. These results are in alignment with our earlier observations that although the mitochondria of the infected cells are affected, reptarenavirus infection does not appear to elicit cytopathic effects [14,32, 33].

Following UGV-1 inoculation, the cells kept between 28 and 32 °C released the highest amount of viral RNA into the media. The quantification of viral NP (the main protein component of the IBs) expression at 3 dpi showed the highest amount of viral antigen in cells kept at 34 °C, whereas cells kept at 32 °C exhibited the highest amount of viral antigen at 6 dpi. Since the infected cells did not show evidence of cytopathic effects, we interpret the release of viral RNA into the growth medium as reflecting virion production, thereby suggesting the 28–34 °C temperature range as optimal for reptarenavirus replication. While freshly UGV-1 inoculated cells kept at temperatures outside this range released lower amounts of viral RNA, the cells passaged 15 days post-UGV-1 inoculation and kept at 24 and 26 °C appeared to release similar levels of UGV-1 RNA compared to those kept in the aforementioned optimal range. We hypothesize this to indicate that, once the infection is established and the majority of cells (if not all) become infected, reptarenavirus replication can continue at lower temperatures, suggesting the poikilothermic nature of the cells. The fact that freshly UGV-1 inoculated cells kept at 24 or 26 °C showed lower viral antigen expression and released less UGV-1 RNA into the medium might indicate impairment of entry and/or the initial steps of infection at these temperatures.

The statistical analyses confirmed a strong positive correlation between the amount of UGV-1 S segment RNA released per cell (as a proxy for virion formation) and NP expression (consistent with IB formation); this was most pronounced for the passaged infected cells. The results showed the antigen expression and the release of viral RNA from UGV-1 infected cells followed a similar trend at most temperatures. However, the freshly inoculated cells kept at 24 or 26 °C released low amounts of viral RNA relative to the amount of NP positive cells/surface area. In addition, the cells kept at 34 °C showed high NP expression levels at 3 dpi, but were not matched by a similar increase in viral RNA release. At 36 °C, the cells showed significantly reduced (approximately 100–500 times less than between 28 and 32 °C) NP expression and little or no RNA release. We hence hypothesize that different factors limiting the completion of the virus' life cycle could be at play at the temperature extremes. Based on the results, it seems evident that virus replication and virion assembly are optimal between 28 and 32 °C. At lower temperatures, we found evidence of dampened viral antigen accumulation in the cultures freshly inoculated with the virus, which could suggest hindrance of the early infection steps, such as endocytosis and release from the endosome. This would be supported by the fact that the infected cells kept at these temperatures following passaging demonstrated stronger NP expression, accompanied by high viral RNA release. At the other end of the temperature range, at 34 °C, we observed an increase in viral antigen expression at 3 dpi but a decline at 6 dpi. This could indicate faster progression of the infection and would align with our former observations that viral RNA release from UGV-1 infected I/1Ki cells declines over time when the cells are kept at 30 °C [32,33]. The decline in NP expression could also relate to the observations of another study where we found the IBs to decline in size and number after reptarenavirus-infected cells were moved from 30–37 °C [30]. On the other hand, the accumulation of viral antigen in the cells could also indicate hindrance of virion assembly, e.g. caused by disturbances in vesicle trafficking. The observations that NP expression appeared to have a faster turnover at higher temperatures would also indicate that efficient virion assembly would require temperatures higher than 26 °C.

We used three different approaches to quantify the NP expression in sections of infected cell pellets; two determined a percentage of NP positive cell area/section (QuPath and UNet generated masks), one the percentage of NP positive cells/section (Cellpose cell segmentation feature implemented in QuPath). While the approaches yielded different absolute values, their results showed a good correlation, indicating that the methods might be complementary for the comparative assessment of immunostained sections. Each method showed limitations in the segmentation/classification of areas or cells (Figs 3 and S17; Table S5). QuPath struggled with detecting negative cytoplasmic areas in weakly stained sections (Fig. S17a,b). QuPath with Cellpose led to underestimating cell counts due to missed large cells or poor segmentation, and occasionally overestimating due to oversegmentation or misidentification of background as cells (Fig. S17b). UNet-generated masks, which are not identifying cells as instances, underestimated nuclear counts if the segmentation was used to extrapolate the latter. Compared to QuPath, UNet masks overestimated total and NP-positive areas, due to the training strategy where labelled cytoplasm with small positive dots resulted in extensive positive regions (Fig. 3 and S17a; Table S5). Therefore, the complementary use of different image analysis strategies was reassuring, and considering their specific limitations, a multimodal digital analysis approach is recommended. While all three methods captured the trend regarding the variation of NP staining/positivity across the different groups, each method used a different strategy to assess a pellet. QuPath, by measuring the pellet surface, offers a relatively simple method of quantification, requiring minimal preparatory work. QuPath with Cellpose integration gives access to a more advanced analysis, allowing to perform cell segmentation and to determine the proportion of positive cells. Benefiting from publicly available models [35,39], this method can yield good results while again requiring only minimal preparatory work (more advanced settings are however accessible if needed). The UNet approach, involving the training of a model from scratch, gives maximal flexibility regarding the choice of parameters that can be assessed (based on the research question), at the expense of requiring a non-negligible amount of preparatory work to annotate examples to train a model. Therefore, we suggest using more than one method for the image analysis, ideally including one evaluating the area of the pellet and a second that measures the proportion of cells.

Temperature is reported to directly influence the host immune response against viral RNA infections [27], e.g. in humans, the type I interferon (IFN) response is more robust at 37 °C, allowing enhanced replication of respiratory viruses at the upper respiratory tract (32–33 °C) [27,28, 43, 44]. It is therefore possible that temperature would affect virus replication through dampening the host’s innate immune response also in the poikilothermic boa constrictors. Interestingly though, despite basking being an essential thermoregulatory behaviour [45], a recent study has shown that snakes with fungal infections do not seek higher basking temperatures which could, potentially, behaviourally induce a febrile response [46]. However, in addition to affecting the immune system, temperature contributes to the outcome of RNA virus infections through affecting the folding, stability and expression of the viral genome, conformation, function and interactions of the viral proteins and transmission efficiency [27]. For instance, we found arthropod (tick) and mammalian (rodent and human) cells cultured at 30 °C to support reptarenavirus replication, even though infection at 37 °C was impaired [30]. These findings, together with the observations of the present study, imply that the effect of temperature on reptarenavirus replication would be mediated through virus- rather than host-associated factors. The fact that reptarenaviruses could replicate at lower temperatures (24 or 26 °C), together with the observations that boa constrictors native to Brazil and Costa Rica harbour different reptarenaviruses [7,8], would indicate that reptarenaviruses tolerate a wide temperature range. It would also align with the hypothesis that their natural hosts could also include other poikilothermic animals, and that even arthropods could contribute to their natural lifecycle.

To conclude, we have demonstrated that boa constrictor-derived cells and reptarenaviruses are both temperature sensitive, with optimal growing temperature ranges that overlap at around 32 °C. Moreover, we observed that reptarenavirus infection does not impact host cell growth. Interestingly, we found that, despite being positively correlated, viral RNA release and NP expression are not equally affected by temperature. In the context of captive snake collections that are housed in terraria at temperatures that usually fluctuate between 22 and 30 °C, these results indicate that alterations in temperature could influence viral loads in reptarenavirus-infected animals and hence also viral spread to co-housed snakes.

## Abbreviations

ANN: artificial neural network
APP: analysis protocol package
BIBD: boid inclusion body disease
BSA: bovine serum albumin
CNN: convolutional neural network
D: day
DAB: 3,3′-Diaminobenzidine
dpi: days post inoculation
FBS: fetal bovine serum
FFFU: fluorescent focus forming unit
GPC: glycoprotein precursor
GPU: graphics processing unit
IBs: inclusion bodies
ICC: immunocytochemistry
IF: immunofluorescence
L: large
MEM: Minimum Essential media
MIMPro: Medical Image Manager Pro
MLP: multi-layer perceptrons
MOI: multiplicity of infection
NP: nucleoprotein
P: passage
PBS: phosphate-buffered saline
PFA: paraformaldehyde
qRT-PCR: quantitative reverse transcription-PCR
QuP: QuPath
QuP+C: QuPath + Cellpose
RdRp: RNA-dependent RNA polymerase
ROIs: regions of interest
RT: room temperature
S: small
TCID50: 50% Tissue Culture Infective Dose
TPB: tryptose phosphate broth
UGV-1: University of Giessen virus 1
WSI: whole slide images
Z: zinc finger protein
